# Sowing Date Regulates Starch Metabolism of Glutinous Rice via Altering High-Temperature Duration to Modify Flavor Compound Composition in Rice Wine

**DOI:** 10.3390/foods15142543

**Published:** 2026-07-18

**Authors:** Jiale Sang, Ying Zhao, Wenlong Nie, Yuchao Peng, Zhiyong Liu, Xiaopeng Wang, Yuchen Zhang, Wei Zhang, Jieyu Luo, Jiangwen Nie, Yu Han, Yong Zhou, Changchun Li

**Affiliations:** 1 Hubei Key Laboratory of Resource Utilization and Quality Control of Characteristic Crops, College of Life Science and Technology, Hubei Engineering University, Xiaogan 432000, China; 2 Xiaogan Academy of Agricultural Sciences, Xiaogan 432000, China; 3 College of Environment and Ecology, Hunan Agricultural University, Changsha 410125, China; 4 Engineering Research Center of Ecology and Agricultural Use of Wetland, Ministry of Education, College of Agriculture, Yangtze University, Jingzhou 434025, China

**Keywords:** sowing date, wine-making glutinous rice, starch synthase, grain quality, rice wine, metabolome

## Abstract

Adjusting sowing date effectively regulates rice growth and grain quality amid climate warming. Here, a field experiment was conducted in the Jianghan Plain to investigate how four wine-making glutinous rice sowing dates (21 May, 28 May, 4 June, and 9 June) regulate starch metabolism and grain quality via temperature, and subsequent effects on sensory quality and metabolome of brewed rice wine. The results showed that delayed sowing significantly reduced grain-filling high-temperature stress duration by up to 60.65% while maintaining stable grain yield. With postponed sowing, key starch synthase activities changed obviously; glutinous rice amylose content decreased from 1.6% to 1.3%, and gel consistency and alkali spreading value increased remarkably. Further fermentation tests indicated that glutinous rice sown on 4 June had the optimal raw material quality, and the brewed rice wine possessed the highest sensory score (29.9% higher than the early-sown treatment T1), as well as abundant volatile compounds. Metabolomic analysis differentiated metabolites across treatments, and correlation analysis verified that high-temperature duration indirectly regulated rice wine quality via grain physicochemical traits. In conclusion, moderate sowing delay alleviates high-temperature stress, optimizes raw grain quality and enhances rice wine flavor. The best sowing time for wine-making glutinous rice here is 4 June.

## 1. Introduction

Rice is one of the most essential staple crops worldwide and plays a pivotal role in food security and the traditional fermented food industry across Asia [1,2]. Glutinous rice, a distinctive rice variety, features a unique endosperm structure with an extremely low amylose content and high amylopectin content. It serves as an indispensable raw material for traditional Eastern foods such as rice wine, yellow rice wine and glutinous rice cakes [3,4]. Rice is rich in a variety of essential nutrients for humans, including starch, high-quality plant protein, dietary fiber, B vitamins, potassium, magnesium, phosphorus and trace minerals. Starch acts as the primary energy source to sustain human daily metabolic activities. Rice protein contains balanced essential amino acids that support human tissue repair and growth. Abundant dietary fiber facilitates intestinal peristalsis and improves digestive health. B vitamins participate in carbohydrate and lipid metabolism, while mineral elements such as potassium help regulate human electrolyte balance and maintain normal cardiovascular function [2,3,5]. The quality of glutinous rice directly determines the fermentation efficiency, flavor profiles and economic benefits of the rice wine industry [4,5,6]. In recent years, driven by consumption upgrading and the rapid expansion of the specialty food market, the market demand for high-quality glutinous rice products has been steadily increasing. Accordingly, higher requirements have been put forward for the yield stability, grain quality and processing suitability of special-purpose glutinous rice cultivars [7]. Against this backdrop, improving the eating and processing quality of glutinous rice grains has attracted growing academic attention, and previous studies have conducted extensive research on the quality formation of glutinous rice and its application in fermentation [8,9,10].

Grain filling is a critical developmental stage that determines the final quality of rice grains, and temperature acts as a core environmental factor regulating the grain-filling process and dry matter accumulation [8,11,12]. As a vital agronomic management practice, sowing date reshapes field light and temperature conditions, thereby modulating grain-filling dynamics and quality formation [13,14]. Appropriate adjustment of sowing date can markedly alter the activities of key enzymes involved in starch synthesis, optimize the accumulation and partitioning of dry matter, and remodel the physicochemical properties of rice grains, ultimately exerting profound effects on the eating and processing quality of glutinous rice [15,16,17,18]. Previous studies have demonstrated that moderately delayed sowing can optimize the temperature regime during the grain-filling stage, effectively reduce grain chalkiness, improve gel consistency and gelatinization properties, and enhance the cooking and processing quality of rice grains [19,20]. In glutinous rice grains, granule-bound starch synthase (GBSS), starch branching enzyme (SBE), soluble starch synthase (SSS) and debranching enzyme (DBE) collectively regulate the synthesis and fine structure of amylose and amylopectin, which directly govern the viscosity, gelatinization temperature and fermentation performance of glutinous rice [21,22]. Meanwhile, physicochemical indices of glutinous rice, including amylose content, alkali spreading value and gel consistency, are significantly correlated with the total acid, reducing sugar, amino acid nitrogen, flavor and sensory quality of brewed rice wine [23,24]. Grain components such as starch and protein are core determinants of the aroma complexity and commercial value of rice wine [25,26].

Although existing studies have clarified the separate correlations among sowing date, starch metabolism and glutinous rice quality, as well as the relationship between rice grain quality and rice wine flavor quality, most current research focuses merely on agronomic studies regarding the independent response of rice quality to single cultivation factors or food science analyses linking grain quality to fermentation indicators of rice wine. To date, few systematic integrated analyses have been reported on the complete regulatory cascade of sowing date regulation–glutinous rice growth and grain quality–physicochemical properties, flavor and metabolome of rice wine, leaving a prominent research gap in interdisciplinary integration. Consequently, the chain mechanism by which sowing date modulates the raw material characteristics of glutinous rice and further affects the formation of flavor substances in rice wine remains poorly understood. This hinders the targeted quality improvement of rice wine-specific glutinous rice cultivars and precise quality regulation in industrial production.

In this study, rice wine-dedicated glutinous rice cultivars were used as experimental materials, and four sowing date gradients were established. We systematically investigated the regulatory effects of sowing date on the activities of key starch synthesis enzymes, dry matter accumulation, yield components and grain quality of glutinous rice. Combined with rice wine fermentation trials, physicochemical detection, sensory evaluation, untargeted metabolomics and volatile metabolomics analyses, we further explored the intrinsic relationship between raw material characteristics of glutinous rice and processing quality of brewed rice wine. The main objectives of this study are as follows: (1) to elucidate the regulatory patterns of different sowing dates on heat stress and the activities of starch metabolism-related enzymes in glutinous rice; (2) to clarify the linkage mechanism among grain physicochemical traits, fermentation properties and flavor metabolism of rice wine; (3) to propose differentiated sowing date strategies for glutinous rice in accordance with the quality requirements of rice wine brewing. This work aims to provide a theoretical basis and technical support for the targeted cultivation and quality improvement of rice wine-specific glutinous rice under the background of ongoing climate warming. From a practical perspective, this study provides a low-cost sowing regulation strategy for wine-making glutinous rice production in the Jianghan Plain and offers a theoretical basis for rice wine enterprises to screen high-quality brewing raw materials under climate warming.

## 2. Materials and Methods

### 2.1. Experimental Site

The field experiment was conducted at the experimental base of Ebao Glutinous Rice Seed Industry Technology Co., Ltd. in Xiaogan City, Hubei Province, China (30.82° N, 113.80° E) in 2025. The study site has a subtropical continental monsoon climate, with an annual average temperature of 15.5–16.2 °C, annual precipitation of approximately 1140 mm, and annual sunshine duration of approximately 2000 h. The soil pH was 6.1, with soil organic carbon of 28.4 g/kg, total nitrogen (TN) of 1.7 g/kg, total phosphorus (TP) of 0.5 g/kg, total potassium (TK) of 11.2 g/kg, available phosphorus (AP) of 21.5 mg/kg, and available potassium (AK) of 139.5 mg/kg.

### 2.2. Experimental Design

Four sowing and transplanting date treatments were set in this study, namely T1 (sown on 21 May, transplanted on 15 June), T2 (sown on 28 May, transplanted on 22 June), T3 (sown on 4 June, transplanted on 29 June), and T4 (sown on 9 June, transplanted on 4 July). Each treatment was repeated three times with a plot area of 28 m^2^ (4 m × 7 m) and a transplanting density of 20 cm × 16 cm. Uniform field pest, weed control and irrigation management consistent with local Jianghan Plain middle rice practices were carried out across all plots. For agrochemical application: Pre-transplant herbicide was used for weed closure; and low-toxic rice insecticides and fungicides were evenly sprayed at tillering and heading periods to control planthoppers, sheath blight and false smut, without high-toxic pesticides. For irrigation: A 3–5 cm shallow water layer was kept from transplanting to tillering; field drying was performed at late tillering; wet–dry alternate shallow irrigation was applied during jointing and grain-filling stages; and irrigation was terminated seven days before grain ripening. Three-stage split fertilization was adopted with urea, superphosphate, potassium chloride, zinc sulfate and silicon fertilizer. Total nutrient inputs were 180 kg ha^−1^ N, 75 kg ha^−1^ P_2_O_5_, 150 kg ha^−1^ K_2_O, 3 kg ha^−1^ Zn fertilizer and 30 kg ha^−1^ silicon fertilizer. All phosphorus, zinc and silicon fertilizers, together with 50% N and 50% K, were applied as basal fertilizer and incorporated 3 days before transplanting; 30% N and 25% K were topdressed as tillering fertilizer 7 days after transplanting; and the remaining 20% N and 25% K were applied as panicle fertilizer at the jointing stage. The local dominant glutinous rice cultivar “Zhennuo”, with a growth period of 132 d and a conventional sowing period in mid-May, was used as the test material. It is an inbred indica glutinous rice bred by the Institute of Food Crops, Hubei Academy of Agricultural Sciences, derived from a cross between Hubei local waxy landrace Zhongxiannuo 1776 (female parent) and Guangxi local landrace Zhendao (male parent) via seven generations of pedigree self-purification, and acquired national new plant variety rights in 2016. It features ~1.3% amylose and an excellent waxy texture, suitable for brewing Xiaogan rice wine and high-grade Baijiu. The narrow sowing intervals are limited by the local narrow suitable sowing window for glutinous rice. The original 7-day interval was planned for all groups, but T4 was advanced from 9–11 June to avoid heavy rain forecasted on 10–11 June, leading to a shorter gap between T3 and T4.

Rice wine was brewed using glutinous rice grains harvested from the four treatments (T1–T4) with commercial honeycomb rice koji, which is widely applied in local rice wine factories. A mass of 500 g of dry glutinous rice was used for a single fermentation replicate, and three replicates were prepared for each sowing treatment, amounting to 1500 g of glutinous rice per treatment. The unified brewing process was as follows: Rice grains were stored for three months and shelled, then soaked in clean water for 8 h and steamed in an electric rice cooker for 1 h. The cooked rice was naturally spread and cooled to 32 °C, mixed with 0.4% rice koji (*w*/*w*) and 120% sterile cold boiled water (*w*/*w*), and sealed with plastic film after subpackaging. Rice koji used in this study was handmade by traditional techniques and purchased from Xiaogan Wangqi’an Company. It is produced from rice flour blended with herbal ingredients such as honeysuckle grass, polygonum hydropiper and verbena, and it is adopted by more than 30 local rice wine manufacturers in Xiaogan. Microbiologically, rice koji acts as a traditional fermentation starter containing *Rhizopus oryzae*, yeast and lactic acid bacteria. During brewing, *Rhizopus oryzae* breaks down rice starch into fermentable monosaccharides, which are further utilized by yeasts and lactic acid bacteria to produce ethanol, organic acids and characteristic flavor metabolites. Fermentation was performed at a constant temperature of 30 °C for 60 h. Before and during fermentation, the operating room and all experimental containers were sterilized by ultraviolet irradiation and high-temperature boiling to avoid microbial contamination. After fermentation, the fermentation substrates were collected, snap-frozen with liquid nitrogen, and stored at −80 °C for subsequent untargeted metabolomics and volatile metabolomics analyses.

### 2.3. Determination of Indicators and Methods

#### 2.3.1. Rice Yield and Yield Components

At the mature stage of glutinous rice, four 1 m^2^ uniform rice sampling points were selected in each plot to determine the actual grain yield after threshing, sun-drying and impurity removal. Six representative rice hills were sampled per plot according to the average tiller number to measure effective panicle number, grain number per panicle, seed setting rate, and 1000-grain weight. Seed setting rate was calculated as follows: Seed setting rate (%) = Number of fully filled grains/Total grain number per panicle × 100%. All rice samples were killed at 105 °C for 30 min and dried to constant weight at 70 °C for subsequent data calculation. All samples were collected avoiding border effects; the same applies hereinafter.

#### 2.3.2. Grain-Filling Temperature and Grain-Filling Rate

A TH20 temperature and humidity recorder (Xianjing Electronic Technology Co., Ltd., Wuxi, China) was fixed at the canopy height of rice plants to automatically record high-temperature duration, daily maximum temperature, daily minimum temperature, and daily high-temperature stress duration (temperature > 35 °C) during the grain-filling stage.

Fifty uniformly flowering panicles were marked at the initial flowering stage of each treatment. From the 10th day after flowering to maturity, 10 panicles were sampled every 7 d per plot using the five-point sampling method. Full grains were screened and dried to constant weight following standard oven-drying procedures. Grain-filling rate (mg·grain^−1^·d^−1^) was calculated by the following formula: Grain-filling rate = (Current 1000-grain weight—Previous 1000-grain weight)/Sampling interval days. The 1000-grain weight and grain-filling rate were calculated based on the dry weight of grains.

#### 2.3.3. Starch Synthesis Key Enzymes, Dry Matter and C/N Content

Rice grain samples during the filling stage were snap-frozen in liquid nitrogen and stored at −80 °C. The activities of granule-bound starch synthase (GBSS), soluble starch synthase (SSS), starch branching enzyme (SBE), and starch debranching enzyme (DBE) were determined using commercial assay kits (Boxbio Biotechnology Co., Ltd., Beijing, China) according to the manufacturer’s instructions.

At maturity, three representative rice plants were sampled per plot and divided into leaf, stem, and panicle tissues. All samples were killed at 105 °C for 30 min and dried to constant weight at 60 °C to determine above-ground dry matter weight. Dry matter accumulation was obtained by direct weighing of dried constant-weight plant samples, expressed as t·ha^−1^. Dried tissue samples were ground and sieved through a 100-mesh sieve. Approximately 5 mg of sieved powder was used for the determination of total carbon (C) and total nitrogen (N) contents using an elemental analyzer (Costech ECS 4010, Costech Analytical Technologies, Valencia, Italy). Each sample was measured in triplicate.

#### 2.3.4. Rice Grain Quality

The main rice quality indicators, including amylose content, alkali spreading value, and gel consistency, were determined by the Agricultural Product Quality and Safety Supervision, Inspection and Testing Center (Wuhan), Ministry of Agriculture and Rural Affairs. Amylose content, alkali spreading value and gel consistency were determined by standard rice quality methods. Briefly, amylose content was quantified via iodine colorimetric spectrophotometry at 620 nm after lipid removal [20]; alkali spreading value was graded by observing kernel dissolution in 1.7% KOH [22]; and gel consistency was measured as the horizontal flow length of rice gel gelatinized in 0.2 mol/L KOH after cooling [24].

#### 2.3.5. Physicochemical Properties of Rice Wine

Total acid content was determined via sodium hydroxide–phenolphthalein titration [8]; reducing sugar content was measured by the DNS colorimetric method [15]; amino acid nitrogen was quantified using formaldehyde–sodium hydroxide titration [21]; and alcohol content was determined by the distillation–alcoholimeter method [23]. The pH value was tested with a PHB-5 pH meter manufactured by Shanghai INESA Scientific Instrument Co., Ltd., with three biological replicates per treatment.

The sugar content (°Brix) of rice wine was detected immediately after the termination of 60 h fermentation using a portable hand-held refractometer (MASTER-T, ATAGO, Tokyo, Japan) with automatic temperature compensation (ATC, 10–30 °C compensation range). The instrument, with a measurement range of 0–32 °Brix and an accuracy of ±0.1 °Brix, was calibrated with distilled water before each measurement, and each sample was determined in triplicate. The initial Brix value of raw glutinous rice from all four treatments was 0 °Brix, since Brix only detects soluble small molecular substances; starch, the main carbohydrate in unfermented rice, cannot be measured without hydrolytic decomposition.

#### 2.3.6. Sensory Evaluation of Rice Wine

Sensory evaluation of fermented rice wine was performed by 31 trained panelists in a standard sensory evaluation room. Four evaluation indices, including color (10 points), fragrance (20 points), taste (20 points), and form (10 points), were assessed to comprehensively evaluate the overall quality of rice wine.

#### 2.3.7. Multi-Omics Determination

The volatile metabolomics analysis of glutinous rice wine was performed by Matewalk Biotechnology Co., Ltd., Wuhan, China, via headspace solid-phase microextraction coupled gas chromatography–mass spectrometry (HS-SPME-GC-MS) with complete instrumental parameters as follows: 120 µm DVB/CWR/PDMS SPME Arrow fiber was adopted; samples were extracted at 60 °C for 15 min and desorbed at 250 °C for 5 min. A DB-5MS capillary column (30 m × 0.25 mm × 0.25 µm) was used with high-purity helium carrier gas at a constant flow rate of 1.2 mL/min. The oven temperature program: hold at 40 °C for 3.5 min, rise to 100 °C at 10 °C/min, rise to 180 °C at 7 °C/min, and finally rise to 280 °C at 25 °C/min and hold for 5 min. Mass spectrometry adopted electron impact (EI) ionization at 70 eV; ion source temperature 230 °C, quadrupole temperature 150 °C, transmission line temperature 280 °C, with selected ion monitoring (SIM) mode. Metabolites were identified by matching a self-built volatile mass spectrum library, retention time, characteristic qualitative/quantitative ions and retention index; compounds with relative odor activity value (rOAV) ≥ 1 were regarded as core flavor substances. The untargeted metabolomics analysis was conducted by Tsingke Biotechnology Co., Ltd., Beijing, China, via ultra-high performance liquid chromatography–tandem mass spectrometry (UPLC-MS/MS). Standard untargeted LC-MS testing workflows, including chromatographic column, mobile phase composition, gradient elution procedure and full-scan MS/MS acquisition parameters, were implemented following the platform’s standardized protocols. Metabolite identification relied on matching primary and secondary fragment ion spectra with HMDB and KEGG compound databases.

For multi-omics data preprocessing and statistics, all metabolite signal data were subjected to log_2_ transformation and unit variance (UV) scaling. Differential metabolites were screened by unified criteria: VIP > 1 combined with Student’s *t*-test *p* < 0.05 for pairwise comparisons; and VIP > 1 combined with ANOVA *p* < 0.05 for multi-group comparisons. Principal component analysis (PCA), orthogonal partial least squares discriminant analysis (OPLS-DA), hierarchical clustering, correlation analysis and KEGG pathway enrichment were completed using R software (version 4.2.1). The 200-times permutation test was carried out to verify OPLS-DA model reliability.

### 2.4. Data Analysis

All experimental data were sorted using Microsoft Excel 2019. One-way analysis of variance (ANOVA) was conducted at *p* < 0.05 using SPSS 26.0 software, and Duncan’s multiple range test was used for post hoc multiple comparisons to separate significant differences among treatments. Correlation analysis and data visualization were conducted using Origin 22.0. For multi-omics datasets, raw metabolite signals were log_2_-transformed and unit variance (UV)-scaled before analysis. Principal component analysis (PCA), orthogonal partial least squares discriminant analysis (OPLS-DA), hierarchical clustering, Pearson correlation and KEGG pathway enrichment analysis were performed using R software (version 4.2.1). A total of 200 permutation tests were implemented to eliminate overfitting risks of the OPLS-DA model.

## 3. Results

### 3.1. Variation in the Duration of High-Temperature Stress During Grain Filling

As shown in Table 1, delaying sowing effectively shortened the cumulative duration and frequency of high-temperature stress (>35 °C) during critical growth stages, including heading and grain filling. The cumulative duration of exposure to temperatures above 35 °C reached 98.19 h in the T1 treatment, which also experienced three episodes of continuous high temperature lasting over three consecutive days (6 days, 7 days and 3 days, respectively). The high-temperature stress duration decreased to 81.83 h and 58.83 h in T2 and T3, accompanied by fewer high-temperature episodes. The cumulative high-temperature duration in T4 was only 38.64 h, representing a 60.65% reduction compared with T1. These results indicate that delayed sowing enables glutinous rice to effectively avoid high temperatures during grain filling and mitigates the adverse impacts of heat stress on panicle and grain development.

### 3.2. Dry Matter Accumulation, Carbon and Nitrogen Partitioning, and Yield Components

As presented in Table 2, no significant differences in total above-ground dry matter weight were observed across the four treatments (*p* > 0.05). Delayed sowing significantly reduced the seed setting rate and 1000-grain weight (*p* < 0.05). The seed setting rate of T1 was markedly higher than that of T3 and T4. T1 and T2 exhibited greater 1000-grain weight than the latter two treatments; no significant difference was found between T1 and T2, while both values were significantly higher than that of T3. The number of effective panicles and grains per panicle showed no obvious variation among all groups.

Although sowing was postponed, no significant difference in actual grain yield was observed among all treatments. In addition, sowing date exerted no significant effects on carbon and nitrogen accumulation in leaves, stems and panicles of glutinous rice.

### 3.3. Activities of Key Starch Synthases and Grain-Filling Dynamics

**Figure 1:**
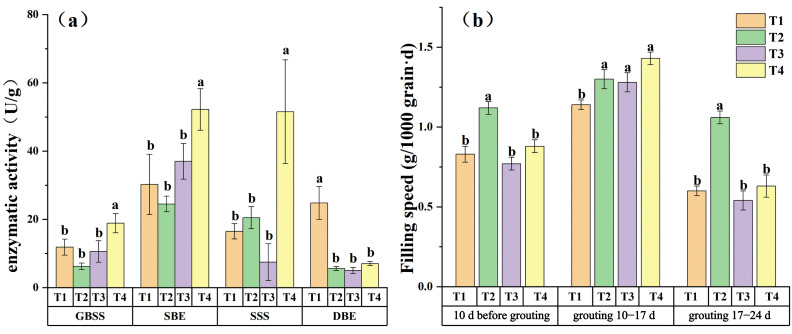
Effects of different sowing dates on activities of key starch synthases and grain-filling rate of glutinous rice. (**a**) Activities of four key starch metabolic enzymes in glutinous rice during grain filling; (**b**) dynamic changes of grain-filling rate of glutinous rice. Different lowercase letters in the same group indicate significant differences (*p* < 0.05); T1 (21 May), T2 (28 May), T3 (4 June), T4 (9 June), the same below.

Variations in ambient temperature during grain filling caused distinct changes in the activities of four key starch metabolic enzymes, namely granule-bound starch synthase (GBSS), soluble starch synthase (SSS), starch branching enzyme (SBE) and debranching enzyme (DBE) (Figure 1a). The activities of GBSS, SSS and SBE in T4 were significantly higher than those in other treatments, while no significant differences were detected among T1, T2 and T3. The activities of GBSS and SBE decreased first and then increased with delayed sowing, with the lowest values observed in T2. The minimum SSS activity was recorded in T3. The DBE activity of T1 was significantly higher than that of T2, T3 and T4, and no significant difference existed among the latter three groups.

The grain-filling rate of all treatments followed a consistent dynamic pattern: slow growth at the early stage, rapid elevation at the middle stage and gradual decline at the late stage (Figure 1b). T2 exhibited an overall superior grain-filling rate. Its filling rate was significantly higher than the other three groups at 10 days and 24 days after flowering, while no significant difference was found among T1, T3 and T4. At 17 days after flowering, the grain-filling rate of T2, T3 and T4 was remarkably higher than that of T1, with no significant differences among T2, T3 and T4.

### 3.4. Effects of Sowing Date on Grain Quality of Glutinous Rice

Sowing date significantly altered three core quality indicators of glutinous rice: amylose content, gel consistency and alkali spreading value (Table 3). Amylose content gradually decreased as sowing was postponed. The amylose content of T1 was 1.6%, which was two percentage points higher than T2 and three percentage points higher than T3 and T4. No significant difference in amylose content was observed among T2, T3 and T4.

Gel consistency and alkali spreading value increased with delayed sowing. T1 had the lowest gel consistency (91.0 mm), which was significantly lower than T2 (93.7 mm). T3 and T4 showed comparable gel consistency, both of which were significantly higher than T1 and T2. The alkali spreading value was identical (4.5) in T1 and T2, representing the lowest level across all groups. T3 and T4 shared the same alkali spreading value (6.0), which was 33.33% higher than that of the first two treatments.

### 3.5. Effects of Sowing Date on Sensory and Physicochemical Quality of Rice Wine

The sensory quality of rice wine was markedly affected by the sowing date of raw glutinous rice (Table 4). No significant difference was found in the color score among all treatments. The aroma score rose first and then declined with delayed sowing, reaching the maximum in T3. The aroma score of T3 was significantly higher than T4, and both were superior to T1 and T2. The variation trend of taste score was consistent with aroma score; T3 and T4 obtained high taste scores that were significantly higher than T1 and T2. In terms of wine body morphology, T1 and T2 scored significantly higher than T3.

The total sensory score ranked as T3 > T4 > T2 > T1. The total score of T3 was approximately 30% higher than that of T1, and T4 also exhibited significantly better sensory performance than T1.

**Figure 2:**
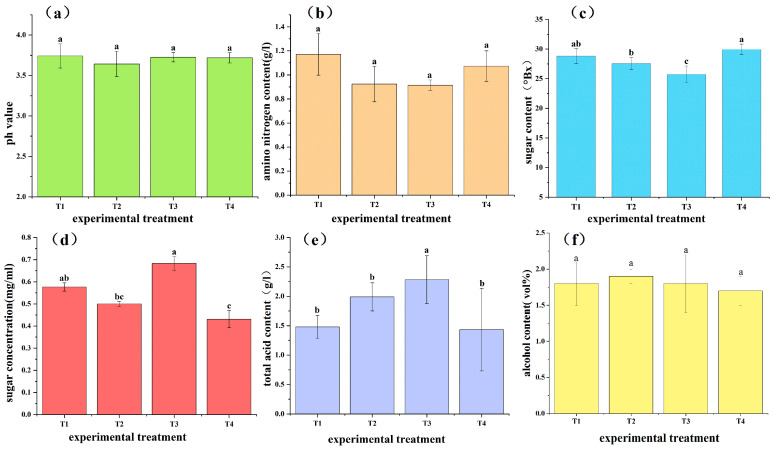
Physicochemical properties of rice wine brewed from glutinous rice under different sowing dates. (**a**) pH value; (**b**) amino acid nitrogen content; (**c**) sugar content; (**d**) sugar concentration; (**e**) total acid content; (**f**) alcohol content. Different lowercase letters in the same group indicate significant differences (*p* < 0.05).

As shown in Figure 2, pH value and amino acid nitrogen content showed no significant differences across treatments, while reducing sugar content, total acid content and sugar degree differed significantly. T3 had the highest reducing sugar content, which was markedly higher than T2 and T4; the reducing sugar content of T4 was significantly lower than that of T1. The total acid content also peaked in T3 and was significantly higher than T1, T2 and T4, with no significant difference among the latter three groups. Sugar degree decreased first and then increased with delayed sowing: T3 had the lowest sugar degree, while T4 presented the maximum value, which was significantly higher than T2 and T3. Alcohol content of all treatments ranged from 1.7 to 1.9 vol%, with no significant differences among treatments (*p* > 0.05).

### 3.6. Analysis of Volatile Metabolites in Rice Wine

#### 3.6.1. Volatile Aroma Compounds (HS-SPME-GC-MS)

**Figure 3:**
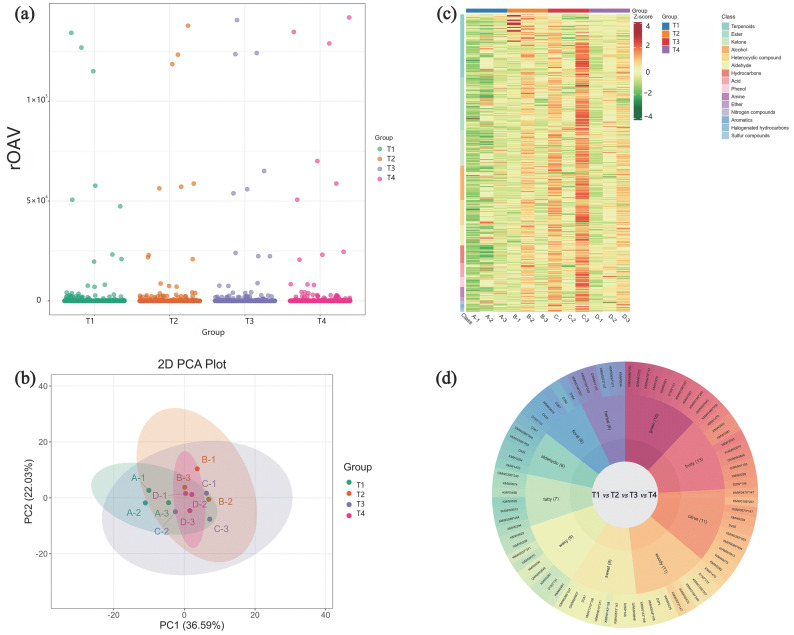
Analysis of volatile aroma compounds in rice wine. (**a**) Relative odor activity value (rOAV) of key aroma compounds; (**b**) two-dimensional principal component analysis (PCA) of volatile metabolites; (**c**) the abundance of volatile compounds; (**d**) classification and aroma characteristics of differential volatile metabolites.

Compounds with relative odor activity value (rOAV) ≥ 1 were defined as key aroma substances. Key aroma compounds were detected in all groups, and their rOAV values gradually increased with delayed sowing, with the highest level in T4, indicating that postponing sowing enhanced the aroma contribution of key flavor compounds in rice wine (Figure 3a).

Principal component analysis (PCA) revealed that the first principal component (PC1) explained 36.59% of the total variance, and all samples were clearly distinguished, demonstrating that changes in rice sowing date substantially altered the composition of volatile metabolites in rice wine (Figure 3b).

The abundance of volatile compounds was the highest in T3, followed by T4 (Figure 3c). These two groups accumulated higher contents of terpenoids, esters, ketones and other typical flavor substances, indicating that delayed sowing facilitated the accumulation of flavor compounds, with T3 showing the optimal effect (Figure 3d). Differential metabolites mainly contributed to fruity and herbal aromas, which constituted the dominant fragrance of rice wine. T4 displayed more prominent fruity and woody notes, which was consistent with the rOAV analysis results and reflected superior flavor quality. The relative content of volatile metabolites is shown in Supplementary File S1.

#### 3.6.2. Untargeted Metabolomics (UPLC-MS/MS)

**Figure 4:**
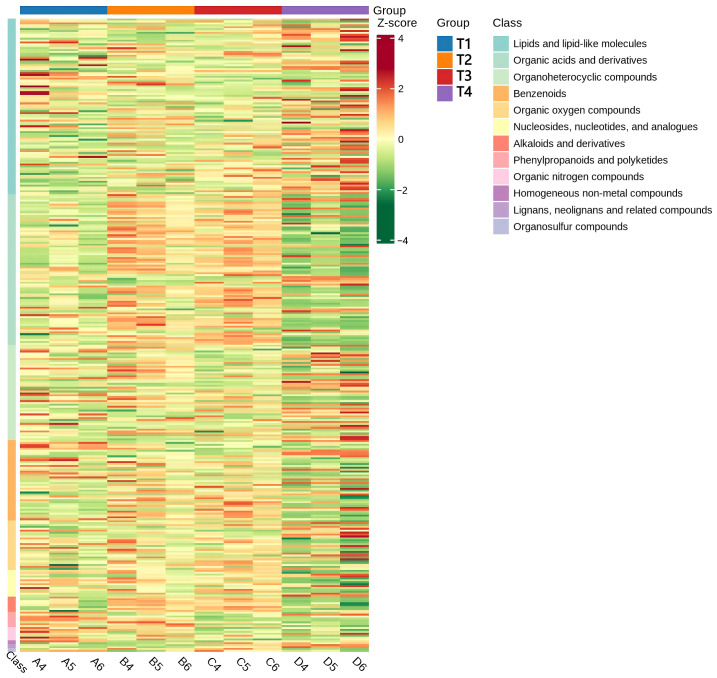
Distribution of major metabolites based on untargeted metabolomics.

The Z-score distribution of major metabolite classes (Figure 4) reflected distinct differences in the relative abundance of various metabolites among the four treatments. Lipids and lipid-like molecules were highly expressed and dominated in T1. Organic acids and their derivatives were the predominant high-abundance metabolites in T2. Organoheterocyclic compounds were markedly enriched in T3, while benzenoids were the major high-expression metabolites in T4.

Other metabolite categories, including alkaloids, lignans and organosulfur compounds, showed low overall expression with minor inter-group fluctuations and did not serve as characteristic components. Distinct grouping was formed based on the abundance of major metabolite classes, and the characteristic metabolic categories of each sample were well differentiated.

### 3.7. Correlation Analysis of Key Indicators

**Figure 5:**
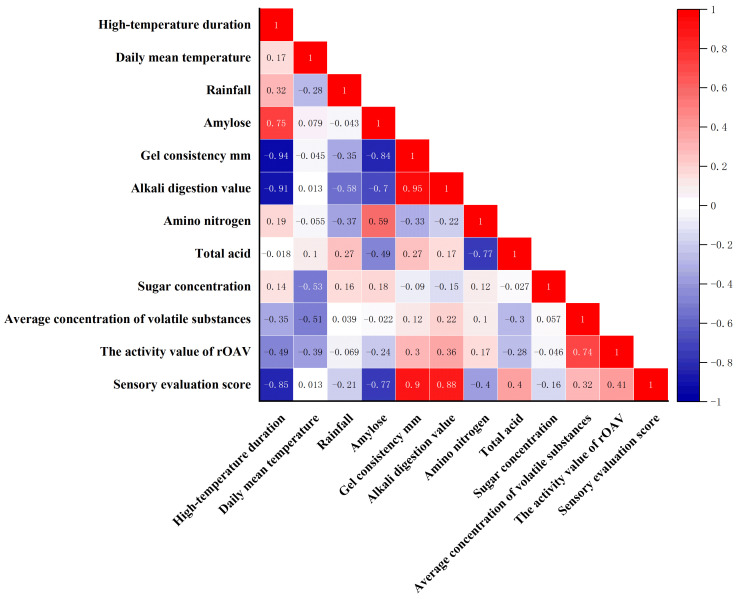
Correlation heatmap of meteorological factors, glutinous rice quality, physicochemical properties and flavor indices of rice wine under different sowing dates.

Pearson correlation analysis (Figure 5) showed that the high-temperature duration during growth had a strong positive correlation with amylose content (correlation coefficient = 0.75), and a strong negative correlation with gel consistency and alkali spreading value. In addition, the high-temperature duration was significantly negatively correlated with the sensory score of rice wine (r = −0.85) and showed weak to moderate negative correlations with the average concentration and rOAV of volatile substances. Meteorological factors such as daily average temperature and rainfall showed weak correlations with other indicators.

In terms of the correlation between grain quality and rice wine sensory properties, amylose content had a strong negative correlation with sensory scores, whereas gel consistency and alkali spreading value were strongly positively correlated with sensory scores (correlation coefficients of 0.90 and 0.88, respectively).

The correlation direction and intensity varied greatly among different indicators. These results confirmed that meteorological conditions during rice growth could substantially regulate grain quality traits, and variations in raw glutinous rice quality would further affect the physicochemical properties and flavor quality of rice wine. A stepwise correlative chain was established among cultivation environment, raw grain quality and finished rice wine quality.

## 4. Discussion

### 4.1. Correlation of Sowing Date-Regulated Grain-Filling Temperature with Growth and Yield Traits of Glutinous Rice

Delayed sowing markedly reduced the cumulative duration and frequency of high-temperature stress (>35 °C) during the heading and grain-filling stages of glutinous rice, with the cumulative high-temperature duration in the T4 treatment decreasing by 60.65% compared with T1. Previous studies have demonstrated that sustained high temperature during grain filling inhibits grain development and reduces 1000-grain weight and seed setting rate [23,26]. Unlike previous studies, the early-sown group (T1) experienced longer duration of high temperature but exhibited a higher seed setting rate and 1000-grain weight. This phenomenon was probably attributed to the fact that the grain-filling stage of late-sown groups fell in autumn, when the overall temperature and light resources were insufficient [25].

In the present study, delayed sowing lowered ambient temperature during grain filling and alleviated heat stress, yet it also led to significant declines in seed setting rate and 1000-grain weight. No significant differences were detected in above-ground total dry matter accumulation, carbon and nitrogen partitioning across plant organs, effective panicle number or grain number per panicle among all treatments. For early-sown rice, despite heat stress during grain filling, sufficient light and temperature conditions facilitated adequate grain filling, thereby maintaining a high seed setting rate and 1000-grain weight. By contrast, late-sown rice escaped high-temperature stress but experienced a shortened effective grain-filling period due to delayed growth progression, which resulted in insufficient grain plumpness and declined yield components [27]. The countervailing effects of these two factors ultimately stabilized the actual yield across treatments.

Collectively, adjusting sowing date reshapes field light and temperature conditions, serving as a pivotal agronomic strategy to balance heat stress mitigation and grain formation for glutinous rice dedicated to rice wine production [28]. This conclusion is consistent with previous reports that growth period regulation enables rice to evade peak high-temperature periods [29].

### 4.2. Regulatory Effects of Sowing Date and Temperature on the Activities of Key Starch Synthases and Grain-Filling Dynamics

Starch constitutes the predominant component of glutinous rice grains. Granule-bound starch synthase (GBSS), starch branching enzyme (SBE), soluble starch synthase (SSS) and debranching enzyme (DBE) collaboratively govern starch synthesis efficiency and molecular structure. Variations in ambient temperature induced by altered sowing dates caused distinct changes in the activities of these four starch metabolic enzymes. The activities of GBSS, SBE and SSS in the latest sowing treatment (T4) were significantly higher than those in other groups, whereas the maximum DBE activity was observed in the early-sown T1 group. The activities of GBSS and SBE exhibited a trend of decreasing first and then increasing with delayed sowing, with the lowest values detected in T2; the SSS activity reached its minimum in T3. The grain-filling rate of all treatments followed a typical dynamic pattern: slow increase in the early stage, rapid elevation in the middle stage and gradual decline in the late stage. T2 presented the overall optimal grain-filling rate, which was significantly higher than that of the other three groups at the early and middle filling stages, indicating that temperature regimes exert differential regulation on starch synthase activities and grain-filling processes.

High temperature during grain filling significantly affects the activities of starch synthesis-related enzymes [30,31], thereby altering the composition and structure of starch [32]. Zhang et al. [21] reported that GBSS is mainly involved in amylose synthesis, SBE and SSS are associated with amylopectin synthesis, and DBE functions to remove redundant branches of amylopectin. Chen et al. [20] found that high temperature regulates the expression of the GBSS gene and further affects amylose synthesis. High temperature upregulates the expression of GBSS, the core enzyme responsible for amylose synthesis, thus elevating grain amylose content. Meanwhile, heat stress suppresses SBE and activates DBE, hindering the branching process of amylopectin and reducing its relative proportion. In addition, sustained high temperature alters the size, shape and compactness of starch granules, which further changes the gelatinization and saccharification properties of glutinous rice grains. These molecular and structural changes jointly explain the rising amylose level under prolonged grain-filling heat [29]. In this study, rice in group T1 was exposed to prolonged high temperature, which increased DBE activity but inhibited the activities of GBSS, SBE and SSS. The combined effects of the four enzymes ultimately resulted in a notably higher amylose content in T1. For late-sown treatments, heat stress was alleviated, the activities of the three major starch synthases increased, and amylose accumulation decreased accordingly. This finding is consistent with previous studies [33,34,35]. Differences in grain-filling rates also explained the variations in 1000-grain weight. The T2 treatment possessed favorable light and temperature conditions that supported a high grain-filling rate and sufficient grain plumpness, contributing to a relatively high 1000-grain weight. Although T3 and T4 exhibited superior enzyme activities, their shortened grain-filling duration and diminished filling rate at the late stage led to reduced 1000-grain weight.

### 4.3. Changes in Grain Physicochemical Quality Mediated by Sowing Date and Its Linkage with Rice Wine Brewing

Amylose content, gel consistency and alkali spreading value are core indices for evaluating the processing quality of glutinous rice [36,37], and they also substantially determine the fermentation performance and final quality of rice wine [38]. In this study, amylose content gradually decreased alongside delayed sowing and reduced grain-filling temperature, with T1 showing a significantly higher amylose level than T2, T3 and T4. By contrast, gel consistency and alkali spreading value continuously increased, and T3 and T4 had notably better performance than early-sown groups. Correlation analysis revealed a strong positive correlation between the high-temperature duration during grain filling and amylose content, as well as strong negative correlations between temperature and gel consistency/alkali spreading value, which established a sequential regulatory chain of sowing date–ambient temperature–grain physicochemical quality.

Numerous studies have proven that starch properties of rice are the fundamental determinant of fermented wine quality [39]. Amylose content and gel consistency are regarded as key indicators for screening rice cultivars specialized for wine brewing [40], and the structural and compositional characteristics of glutinous rice starch directly affect the sugar content, alcohol content and taste of rice wine [41]. High ambient temperature is known to increase amylose content in rice grains [42]. The high amylose level in early-sown T1 rice reduced grain viscosity and impaired its suitability for rice wine fermentation. Conversely, late-sown glutinous rice had lower amylose content and higher gel consistency and alkali spreading value, which improved glutinousness and processing adaptability to match the raw material requirements of rice wine production. Furthermore, starch structure profoundly affects starch degradation efficiency during fermentation [43]. The optimized starch properties of late-sown glutinous rice were more susceptible to utilization by fermentative microorganisms, laying a solid material foundation for the subsequent formation of flavor substances in rice wine [44].

### 4.4. Effects of Raw Grain Quality on Physicochemical Properties, Sensory Attributes and Metabolomic Profiles of Rice Wine

#### 4.4.1. Physicochemical and Sensory Quality of Rice Wine

Sowing date altered the intrinsic quality of glutinous rice raw materials and thereby exerted pronounced impacts on multiple quality traits of rice wine. No significant differences in pH value and amino nitrogen content were observed across all treatments, while reducing sugar content, total acid content and sugar degree varied remarkably. T3 yielded the highest levels of reducing sugar and total acid. The overall sensory scores ranked in the order of T3 > T4 > T2 > T1; specifically, the total sensory score of T3 was approximately 30% higher than that of T1, with prominent advantages in aroma and taste, while only a slight decline was detected in its wine body morphology score.

Consistent with previous studies on rice wine fermentation, the protein and starch components of glutinous rice are closely correlated with the accumulation of amino acids and organic acids in fermented products [45]. The characteristics of raw grains predetermine the abundance of metabolic substrates during fermentation, and the contents of total acid and total sugar dynamically change along with microbial metabolism [46]. The early-sown T1 glutinous rice had high amylose content and poor glutinousness, which led to insufficient conversion of fermentation substrates, low levels of reducing sugar and total acid, and weak aroma and taste, corresponding to the lowest sensory score. The T3 raw material possessed optimal physicochemical properties, and its starch was readily degraded by fermentative microorganisms, resulting in massive accumulation of organic acids and reducing sugars and superior comprehensive sensory quality. Despite the excellent grain quality of T4, insufficient substrate supply caused by reduced grain plumpness slightly compromised the overall quality of its brewed rice wine compared with T3.

#### 4.4.2. Volatile Flavor Substances and Untargeted Metabolome

Volatile flavor substances are the core contributors to the flavor profile of rice wine, and relative odor activity value (rOAV) is a reliable index for assessing the contribution of key aroma compounds. T3 exhibited the highest total abundance of volatile metabolites, which can be explained by coordinated physiological and fermentative metabolic mechanisms. Moderately reduced heat stress during T3 grain filling balanced the activities of GBSS, SBE and SBE, producing grains with medium amylose concentration and superior gel consistency. Moderate amylose was readily hydrolyzed by *Rhizopus oryzae* into abundant fermentable sugars; meanwhile, T3 maintained sufficient accumulation of grain proteins and lipids, two critical precursor pools for aroma generation. In the subsequent fermentation system, amino acid catabolism and fatty acid β-oxidation pathways were simultaneously strongly upregulated: proteolysis released large amounts of free amino acids, which were further transformed into aldehydes and alcohols via transamination and deamination, while lipid decomposition generated diverse fruity esters. The synergistic activation of these two core flavor biosynthetic pathways jointly elevated the total volatile metabolite abundance of T3 rice wine [34,36,41]. When T4 entered the grain-filling stage in autumn, cold dew wind and relatively low temperatures reduced the activities of relevant synthetic enzymes in grains, resulting in inadequate total accumulation of core flavor precursors, including proteins and lipids. Limited supply of carbon substrates during fermentation further suppressed the formation of flavor compounds derived from amino acids and fatty acids. Consequently, although several individual aroma compounds in T4 exhibited high rOAV values, the overall abundance of volatile metabolites remained lower than that of T3 [42,43,44]. Principal component analysis (PCA) revealed clear discrimination among volatile metabolomic profiles of rice wine from different sowing date treatments, demonstrating that variations in raw grains thoroughly reshaped the flavor characteristics of the final product. Untargeted metabolomics further illustrated distinct classification of characteristic metabolites across groups: lipids and lipid-like molecules dominated in T1, organic acids and their derivatives were enriched in T2, organoheterocyclic compounds were the predominant metabolites in T3, and benzenoids were the signature components in T4. The divergent metabolomic profiles further confirmed that raw grain differences drove metabolic differentiation during fermentation.

The composition of flavor substances is a vital criterion to distinguish rice wine produced with different raw materials and processing techniques [47]. Multiple omics studies have verified that raw material variations significantly alter the volatile and non-volatile metabolomes of sweet rice wine, with esters and alcohols serving as the characteristic flavor compounds [48]. The starch and lipid contents of glutinous rice are positively correlated with the accumulation of volatile aroma substances in rice wine. The late-sown glutinous rice possessed optimized starch structure and altered composition of flavor precursors. Under the metabolism of core fermentative microorganisms such as yeasts and lactobacilli, more esters and terpenoids were synthesized, forming richer flavor layers [49]. The distinct categories of non-targeted metabolites among groups also supported the viewpoint that raw material sources and quality remodel metabolic pathways of fermentative microorganisms, ultimately generating differential metabolite profiles.

### 4.5. Integrated Regulatory Cascade

Collectively, this study fully elucidated a hierarchical regulatory cascade: sowing date → grain-filling temperature → activities of key starch synthases → grain quality of glutinous rice → physicochemical properties, sensory attributes and flavor metabolome of rice wine. The intrinsic mechanisms of this cascade are summarized briefly: First, varied sowing dates shift grain-filling phenology and thermal conditions. Early-sown T1 undergoes prolonged heat stress over 35 °C; moderately delayed T3 gains mild temperatures suitable for starch synthesis, while the latest T4 has overly cool conditions limiting dry matter accumulation. Second, high temperature inhibits GBSS, SSS and SBE but boosts DBE activity, raising amylose proportion. T3 balances enzyme levels to form ideal starch traits, yet T4 has ultra-low amylose alongside reduced grain weight and insufficient protein/lipid flavor precursors. Third, starch structure determines saccharification by *Rhizopus oryzae*. T3′s moderate amylose is fully converted to fermentable carbon, with rich grain precursors activating amino acid and lipid oxidation pathways to generate abundant volatiles. In contrast, high-amylose T1 has incomplete saccharification, and highly branched starch in T4 restricts metabolic flux, even with good gelatinization. Overall, sowing date modulates grain-filling heat stress, reprograms starch synthase activity and grain precursor storage, and further alters microbial metabolism during fermentation, resulting in divergent rice wine quality. This multi-stage causal chain fully supports the proposed regulatory framework linking field management to rice wine flavor [37,45,47]. Correlation analysis statistically validated this multi-level linkage: the high-temperature duration showed a strong negative correlation with the sensory score of rice wine (r = −0.85). Amylose content was strongly negatively correlated with sensory scores, while gel consistency and alkali spreading value exhibited strong positive correlations with sensory scores. The established multi-level regulatory chain also delivers operable practical guidance: local farmers can adopt moderate delayed sowing on 4 June to balance grain yield and raw grain quality, while rice wine manufacturers can evaluate raw material brewing performance by monitoring grain-filling high-temperature duration to stabilize the flavor quality of fermented products.

## 5. Conclusions

Delayed sowing effectively helps glutinous rice dedicated to rice wine production evade high-temperature stress during the grain-filling stage. Although it moderately reduces seed setting rate and 1000-grain weight, the actual grain yield remains stable. Temperature variations induced by adjusted sowing dates regulate the activities of key starch synthases, reduce amylose content and improve gel consistency and alkali spreading value of glutinous rice, thereby optimizing the quality of brewing raw materials. Differences in raw grain quality are further transmitted throughout the fermentation process and significantly alter the physicochemical indices, sensory performance and metabolite composition of rice wine. Overall, rice wine brewed from glutinous rice sown on 4 June achieves the best comprehensive quality. Significant sequential correlations exist among grain-filling high-temperature duration, grain physicochemical traits and rice wine quality. Under the background of global climate warming, moderate delay of sowing date is a feasible agronomic practice to stabilize rice yield, optimize raw grain quality and enhance the flavor of rice wine. Sowing on 4 June, as proposed in this study, can be adopted as the optimal cultivation scheme for rice wine-specialized glutinous rice in this region. The proposed 4 June sowing scheme can be directly applied to wine-making glutinous rice production in this region.

## Figures and Tables

**Table 1:** Duration and frequency of high-temperature stress (>35 °C) during heading and grain-filling stages of glutinous rice.

Experimental Treatment	Initial Heading Stage (10%)	Full Heading Stage (80%)	After Full Heading Stage 20 d	The Length of > 35 °C (h)	Continuous 3 d > 35 °C Times, Days
T1	19 August	27 August	17 September	98.19	3 (6 d, 7 d, 3 d)
T2	27 August	3 September	23 September	81.83	2 (3 d, 7 d)
T3	5 September	9 September	29 September	58.83	2 (6 d, 3 d)
T4	8 September	13 September	3 October	38.64	2(3 d, 3 d)

**Table 2:** Effects of different sowing dates on dry matter accumulation, carbon and nitrogen contents and yield components of glutinous rice.

Experimental Treatment		T1	T2	T3	T4
Total dry matter (t·ha^−1^)		16.5 ± 0.4 a	16.3 ± 0.7 a	16.0 ± 0.2 a	15.6 ± 0.5 a
Seed set propagation coefficient (%)		86.4 ± 1.7 a	83.1 ± 3.6 a	77.2 ± 1.5 b	77.9 ± 1.2 b
Grain number per panicle		203.5 ± 48.9 a	183.0 ± 47.4 a	202.3 ± 35.4 a	193.7 ± 36.4 a
Effective panicles (10^4^·ha^−1^)		347.5 ± 44.8 a	357.5 ± 57.8 a	341.7 ± 46.8 a	327.8 ± 34.1 a
1000-grain weight (g)		23.6 ± 1.6 a	24.2 ± 2.1 a	21.4 ± 1.3 b	22.7 ± 1.2 ab
Grain yield (t·ha^−1^)		9.2 ± 0.4 a	9.2 ± 0.3 a	8.9 ± 0.3 a	8.8 ± 0.2 a
N contents (%)	stem	1.9 ± 1.4 a	2.2 ± 1.0 a	2.1 ± 0.4 a	2.2 ± 0.2 a
	leaf	2.1 ± 0.9 a	2.2 ± 0.5 a	3.6 ± 0.6 a	3.1 ± 1.1 a
	ear	2.9 ± 0.5 a	2.7 ± 1.2 a	2.7 ± 0.5 a	3.2 ± 0.3 a
C contents (%)	stem	15.7 ± 0.3 a	18.7 ± 3.3 a	18.4 ± 0.5 a	17.8 ± 0.2 a
	leaf	13.6 ± 2.3 a	17.3 ± 2.9 a	16.5 ± 1.5 a	18.1 ± 4.9 a
	ear	27.7 ± 0.9 a	26.2 ± 2.5 a	26.2 ± 1.2 a	26.2 ± 1.2 a

Different lowercase letters in the same group indicate significant differences (*p* < 0.05). Values are average (±SE) of the three replicates (*n* = 3).

**Table 3:** Effects of different sowing dates on grain quality traits of glutinous rice.

Quality Indicators	T1	T2	T3	T4
Amylose (%)	1.6 ± 0.1 a	1.4 ± 0.1 b	1.3 ± 0.1 b	1.3 ± 0.1 b
Gel consistency (mm)	91.0 ± 1.0 c	93.7 ± 1.5 b	99.3 ± 1.2 a	100.0 ± 0.0 a
Alkaline extinction level	4.5 ± 0.1 b	4.5 ± 0.1 b	6.0 ± 0.0 a	6.0 ± 0.0 a

Different lowercase letters in the same group indicate significant differences (*p* < 0.05). Values are average (±SE) of the three replicates (*n* = 3).

**Table 4:** Sensory evaluation scores of rice wine brewed from glutinous rice under different sowing dates.

Experimental Treatment	Color (Full Score 10 Points)	Fragrance (Full Score 20 Points)	Taste (Full Score 20 Points)	Form (Full Score 10 Points)	Total Score (60 Out of 60)
T1	7.6 ± 1.5 a	12.4 ± 3.5 d	10.2 ± 3.1 c	8.8 ± 0.8 a	39.1 ± 5.0 d
T2	7.9 ± 1.3 a	14.5 ± 3.2 c	12.8 ± 3.8 b	8.6 ± 1.0 a	43.9 ± 4.8 c
T3	8.2 ± 1.1 a	17.5 ± 1.8 a	17.5 ± 2.0 a	7.5 ± 1.4 b	50.8 ± 3.0 a
T4	8.3 ± 1.2 a	16.0 ± 2.1 b	16.0 ± 3.1 a	8.2 ± 1.1 a	48.5 ± 3.9 b

Different lowercase letters in the same group indicate significant differences (*p* < 0.05). Values are average (±SE) of the three replicates (*n* = 3).

## Data Availability

The original contributions presented in this study are included in the article/Supplementary Materials. Further inquiries can be directed to the corresponding authors.

## Supplementary Materials

The following supporting information can be downloaded at: https://www.mdpi.com/article/10.3390/foods15142543/s1. Table S1: Relative content of volatile metabolites.
